# Network structure-based decorated CPA@CuO hybrid nanocomposite for methyl orange environmental remediation

**DOI:** 10.1038/s41598-021-84540-y

**Published:** 2021-03-03

**Authors:** Dina F. Katowah, Sayed M. Saleh, Sara A. Alqarni, Reham Ali, Gharam I. Mohammed, Mahmoud A. Hussein

**Affiliations:** 1 Department of Chemistry, Faculty of Applied Science, Umm Al-Qura University, P.O. Box 16722, Makkah, 21955 Saudi Arabia; 2 Department of Chemistry, College of Science, Qassim University, Buraidah, 51452 Saudi Arabia; 3 Chemistry Branch, Department of Science and Mathematics, Faculty of Petroleum and Mining Engineering, Suez University, 43721 Suez, Egypt; 4 Department of Chemistry, College of Science, University of Jeddah, Jeddah, Saudi Arabia; 5 Department of Chemistry, Faculty of Science, Suez University, 43518 Suez, Egypt; 6 Department of Chemistry, Faculty of Science, King Abdulaziz University, Jeddah, 21589 Saudi Arabia; 7 Polymer Chemistry Lab, Chemistry Department, Faculty of Science, Assiut University, Assiut, 71516 Egypt

**Keywords:** Chemistry, Environmental chemistry, Materials chemistry, Physical chemistry

## Abstract

A unique network core–shell hybrid design-based cross-linked polyaniline (CPA), which was coated with CuO nanoparticles (NPs) and decorated with nitrogen-doped SWCNT/GO/cellulose N-SWCNTS-GO-CE, has been fabricated using the oxidative polymerization technique. This hybrid nanocomposite shows excellent photocatalytic degradation and an acceptable adsorption capability for Methyl Orange (MO) dye in aqueous solutions with a very slight effect for the N-SWCNTS-GO-CE CuO component. The prepared nanocomposites were used for the removal of a carcinogenic and noxious dye, Methyl Orange, from aqueous samples under various adsorption conditions. Approximately 100% degradation of 10 mg/L of Methylene orange dye was observed within 100 min at pH 6.0 using 50 mg/L CPA/N-SWCNTS-GO-CE/CuO nanocomposite under UV radiation. Additionally, significant factors were investigated on the degradation process including the contact time, MO initial concentration (*C*_*i*_), solution pH, and dosage of the CuO nanocomposite. All investigated experiments were performed under UV radiation, which provided significant data for the MO degradation process. Furthermore, the recovery of the nanocomposite was studied based on the photocatalytic process efficiency. The obtained data provide the high opportunity of reusing CPA/N-SWCNTS-GO-CE/CuO nanocomposite for numerous photocatalytic processes. The CPA/N-SWCNTS-GO-CE/CuO nanocomposite was prepared via chemical oxidative copolymerization of polyaniline (PANI) with p-phenylenediamine (PPDA) and triphenylamine (TPA) in the presence of N-SWCNTS-GO-CE and CuO NPs. The morphology, structure and thermal properties of the CPA/N-SWCNTS-GO-CE/CuO nanocomposite were investigated using various techniques, including FTIR, XRD, RAMAN, SEM, MAP, EDX, TEM, TGA and DTG. Therefore, CPA/N-SWCNTS-GO-CE/CuO nanocomposite can be effectively used as a convenient and reusable adsorbent to remove hazardous dye from wastewater.

## Introduction

Harmful and toxic chemicals, pigments, and dyes increase pollution, can cause dangerous effects, and are predicted to increase in quantity in the coming years^1,2^. Most of the dyes are toxic, persistent and non-biodegradable. It causes serious impact on the environment and adversely affects the life of animals and aquatic species. Based on the chemical structure, dyes are classified into three types i.e. anionic (or acid, reactive and direct dye), cationic (basic dyes) and non-ionic dyes (dispersed dyes)^3^. Methyl orange (MO) is one of the azo dyes that are often used as textile dyes and have different structure which depends on the acidity. Non-biodegradable methyl orange can produce several environmental pollution problems by releasing toxic and carcinogenic compounds in the waters even at low concentrations and results in induced lesions and cancers^4^. MO also famous are used in manufacturing and production of printing paper, and act as pH-indicator that convert its colour while protonated is another well-known hydrophobic anion. MO are the one of the compound that categorized in stable compound with shows a very low of biodegradability and its dissolved in water contact but have a high percentage of difficulty to eliminate from water bodies by using treatment such as purification process of water within industry or by treatment methods^5^. Studies have been developed for dye elimination from aqueous environments, including diverse methods such as physical, biological, and chemical methods^6^. Adsorption methods are considered the most economical process to eliminate water-soluble toxic and dangerous substances. There are varied adsorbent substances such as clays, biomass, wool fibres, biopolymers, and activated carbon, which have been utilized in water-refining methods. Among conducting polymers, poly(aniline-co-p-phenylenediamine) has attracted a lot of attention due to its simple composition, good electrochemical properties, chemical stability, and hopeful applications in many fields^7^. However, the evolution of CPA properties is challenging. Many researchers have examined the properties of CPA using appropriate materials and different synthetic processes^8^. PANI based nanocomposite materials have been utilized as an efficient sorbent for the photocatalytic degradation of different types of organic pollutants as reported in previously in the literature^9–13^. Cross-linking may be suitable for developing electrical and electrochemical properties of the conductive polymers. Yang et al. prepared cross-linked PANI composites by the in situ chemical oxidative polymerization of aniline in the presence of triphenylamine (TPA) and p-phenylenediamine (PPDA)^14^. Until now, there is no study on the utilization of cross-linked PANI for dye removal. Inorganic nanofiller/polymers are new types of polymeric materials, where inorganic nanofillers are incorporatedinto the polymer matrix^15^. These materials combine the advantages of both components. Among various transition metal oxides, e.g., Fe, Ni, Zn, and Cu, the synthesis of CuO is a significant issue of study. The nanostructure of CuO is especially important because it displays the electron transfer at the lower potential, is non-toxic and is comparatively simple to synthesize with various morphologies and dimensions^16,17^. As a p-type semiconductor, CuO is chemically stable, abundant and inexpensive^18,19^. CuONPs are significant due to their numerous applications in gas sensors^20^, biosensors^21^, super-capacitor electrodes^8^, batteries^18^, heterogeneous catalysts^22^, photocatalysis^23–25^, solar cells^26^ and antimicrobial materials^27^.Graphene (G) and carbon nanotubes (CNTs) are two of the most fabricated nanomaterials with unique chemical, electronic, optical, electrochemical and mechanical properties. Due to the unusual properties of the two types of carbons, their large surface area, and very effective electrocatalytic behaviour,their combination is the most attractive candidate for environment treatments^28,29^. The use of nanocellulose as reinforcement in nanocomposites has been considered a prevalent study point. In addition to many features of nanocellulose, such as its low density, low cost, low energy consumption, renewability, biodegradability, high appointed features, and comparatively perfect surface reactivity, nanocellulose displays better features as a reinforcing phase in nanocomposites than micro- or macro-cellulose composites. The tailorability, processability, and design flexibility of nanocellulose polymer composites enable comprehensive exploitation in the packaging, automotive, electronics, and biotechnology industries, among others^30^.

Water contamination due to chemical compounds from industrial sources is a heavy source of environmental pollution. The main ingredients of these contaminants involve dyes, heavy metals, pesticides, detergents, organic compounds and phenol derivatives^31^. Organic dyes are considerably exhausted in many industrial applications such as coating, photographic, textile dyeing and various types of photo-chemical processes in industry. Most of the dyes used as colouring materials are poisonous to aqueous organisms^32^. Enormous efforts have been spent by many scientists to achieve appropriate treatment protocols to remove contaminants from wastewater that originates from diverse industrial sources^33^. Numerous techniques were utilized for dye removal from wastewater, including adsorption systems^31^, membranes^34^ and photocatalytic degradation^35^. The degradation of photocatalytic-based metal oxide nanocatalysts is an advanced oxidation process for dye molecules^36^. This technique was used to remove dye molecules from polluted aqueous solution. The photodegradation procedure applies in the presence of UV radiation and significant metal oxide catalyst^37^. Recently, extraordinary developments have been applied in the dye contaminant photodegradation under ultraviolet light^38,39^. Among numerous metal oxides, CuO is one of the appropriate catalysts for this processdue to its photocatalytic activity, reversibility, non-toxicity and physicochemical properties^40–42^. In our previous work, we prepared variable types of polymer nanocomposites with amazing properties: sensors, conductivity, and coating^43–46^. As a continuation of our previous work, the present work aims to fabricate a nanocomposite of cross-linked CPA with N-SWCNTS-GO-CE and CuO NPs by chemical oxidative copolymerization as effective substances for environmental treatment. The CPA/N-SWCNTS-GO-CE/CuO nanocomposite was applied and evaluated as a potential sorbent to remove MO dye from aqueous solutions. The dye concentration, contact time on the adsorption procedure and effect of pH were examined.

## Experimental

### Materials

p-phenylenediamine (PPDA) was obtained from PDH in UK. Aniline (Ani) was purchased from Shanghai Chemical Reagent Co. Ammonium persulfate (APS) was received from Acros Organics. Triphenylamine (TPA) was obtained from Janssen Chimica (Belgium). Copper oxide (CuO), nitric acid (HNO_3_), sulfuric acid (H_2_SO_4_), urea, ammonia solution, NaOH, ethanol, and cellulose were purchased from Sigma-Andrich (USA). Graphene and SWCNTs were purchased from the XFNANO Advanced Materials Supplier INC. (China). All chemicals and reagents in the investigations were used as obtained. Deionized water was used throughout the examinations.

### Instrumentation and techniques

Field emission scanning electron microscopy (SEM Model Quanta 250 FEG, with 30-kV accelerating voltage at 14 × magnification and up to 1,000,000 resolution for Gun.1n) was utilized to examine the morphological features of the produced materials. High-resolution transmission electron microscopy (HR-TEM) (EM-2100, at 25 × magnification and 200 kV) was also utilized to examine the morphological features of these new materials. Fourier transform infrared (FTIR) spectra were displayed by a JASCO spectrometer over the range of 4000–300 cm^−1^ and used to obtain information on the functional groups of the studied samples. X-ray diffraction (XRD) of the NC crystallinity was examined by using a Bruker model D8 to study the fundamental structures of these nanocomposites. The instrument include reflectometry, high-resolution diffraction, in-plane grazing incidence diffraction (IP-GID), small-angle X-ray scattering (SAXS), and residual stress and texture investigations. Raman spectroscopy measurements were performed by using a Raman spectrometer (Lab. RAM-HR Evolution Horiba Co.) with a single visible spectrometer, which was equipped with an air-cooled open electrode 1024 × 256 pixel CCD detector, a 532 nm He-Cd laser with 1800 grating (450–850 nm), and a 10% ND filter, using an acquisition time of 5 s, 5 accumulations without spike filter and delay time, and a 100 × objective. To detect the absorbance of light wavelengths, the UV–visible recording spectrophotometer Shimadzu-Japan was used. Thermal analyses were detected in the form of TGA and DTG measurements using Shimadzu DTA-50 and TGA-50 systems at a heating rate of 10 °C/min in air. The thermal performance was examined to define the degradation temperature of the NC and their thermal stabilities.

### Fabrication process

#### Synthesis of GO

GO was prepared according to a modified Hummers’ method^47^.

#### Synthesis of OXSWCNTs

According to the literature, the OXSWCNTs were set up^46^. First, 100 mg of SWCNTs were scattered in a mixture (1:3 v/v) of HNO_3_ (70%) and H_2_SO_4_ (96%).Then, they were ultrasonicated for 4 h. Next, for 2 h at 80 °C, the suspension was refluxed in an oil bath with attractive blending. At that point, deionized water (DIW) was used to dilute the received mixture and dialysed in DIW until the washing arrangement demonstrated pH > 5. Finally, OXSWCNTs were filtered and dried in a stove at 60 °C.

#### Synthesis of the N-SWCNTs/GO/cellulose hybrid nanofiller

OXSWCNTs/GO 0.3 g was scattered in DIW100 mL and stirred at 90 °C for 2 h. At that point, 25% aqueous ammonia solution and 0.9 g urea were included and consistently stirred for 12 h at 90 °C. First, because of the formation of the OXSWCNTs/GO hybrid, which dissolved when surplus ammonia was added, the above solution became turbid. Then, in vacuum for 12 h at 90 °C, the transparent solution was evaporated. Cellulose was dissolving in 100 mL H_2_O and 10 g of NaOH. Next, under continuous stirring for another 12 h, the two mixtures were combined at 90 °C. Finally, using ethanol, the final product was washed and dried at 200 °C for 7 h.

#### Synthesis of cross-linked PANI (CPA)

The required pure cross-linked PANI was synthesized as reported in the literature^48,49^.

#### Synthesis of CPA and CPA/N-SWCNTs-GO-CE/CuO nanocomposites

The chemical copolymerization of cross-linked PANI with N-SWCNTs-GO-CE and CuO was achieved as follows:

In a 250-mL three-neck flask consisting of 120 ml of 0.5 M H_2_SO_4_, 5% N-SWCNTs-GO-CE was added. Next, for 2 h at room temperature, the mixture was ultrasonicated. Then, 5% CuO, 1.8626 mL of (doubly distilled) ANI, 0.043256 gm of PPDA and 0.049064 gm of TPA were added. The mixture was ultrasonicated for 30 min. Afterwards, the solution was cooled in an ice bath with continuous stirring at 0–4 °C. Then, 5 g of a pre-cooled solution of ammonium persulfate (APS) dissolved in 0.5 M H_2_SO_4_ 40 mL and added dropwise into the previous solution in approximately 30 min with consistent stirring at 0–4 °C under nitrogen N_2_ atmosphere for 24 h to maintain the polymerization. Next, using ultracentrifugation, the resulting precipitate was collected. Then, it was washed with DIW numerous times until the filtrate became colourless. Finally, the black fine powder was dried at 60 °C for 24 h. This experiment was repeated for comparison; the pure cross-linked PANI was prepared using an identical method without N-OXSWCNTs-GO-CE and CuO NPs.

### Batch method for dye removal

Batch experimentation was afforded to obtain the optimum parameters for the degradation of methyl orange in the presence of the nanocomposite of CPA/N-SWCNTS-GO-CE/CuO as the photocatalyst. The effects of significant variables such as the contact time, pH, nanocomposite dose and initial concentration of methyl orange MO in the medium were exhaustively investigated. To clarify the parameters of the effective reaction, we adjusted one variable at a time. Here, in batch condition, the experiments were conducted with 100 mL 10-mg/mL MO solution in a beaker (250 mL). In the dark, the dye solution with a colloidal suspension of the specified nanocomposite was mixed for 10 min with a magnetic stirrer, and the net solution was centrifuged (4400 rpm/15 min). By using a UV–Vis spectrophotometer, the resulting supernatant was examined. The dye degradation rate was studied using the following equation^50^:

$$ R = \frac{{{\text{C}}_{0} - {\text{C}}_{t} }}{{{\text{C}}_{0} }} \times 100 $$

where R is the efficiency rate of dye removal, C_0_ is the MO dye initial concentration (mg/L), and C_t_ is the concentration of MO dye after the adsorption process per time t (min).

## Results and discussion

In this study, we aimed to fabricate a nanocomposite of cross-linked CPA with N-SWCNTS-GO-CE and CuO NPs. CPA/N-SWCNTS-GO-CE/CuO nanocomposites were prepared using the previous method, which is the chemical oxidative copolymerization of PANI with p-phenylenediamine PPDA and triphenylamine TPA, but in the presence of N-SWCNTS-GO-CE and CuO. Their morphology, structure, and thermal properties of the nanocomposites were examined via several techniques: SEM, TEM, FTIR, XRD, TGA, DTG, RAMAN and dye removal measurements. The result was as follows.

### Surface morphology

**Figure 1:**
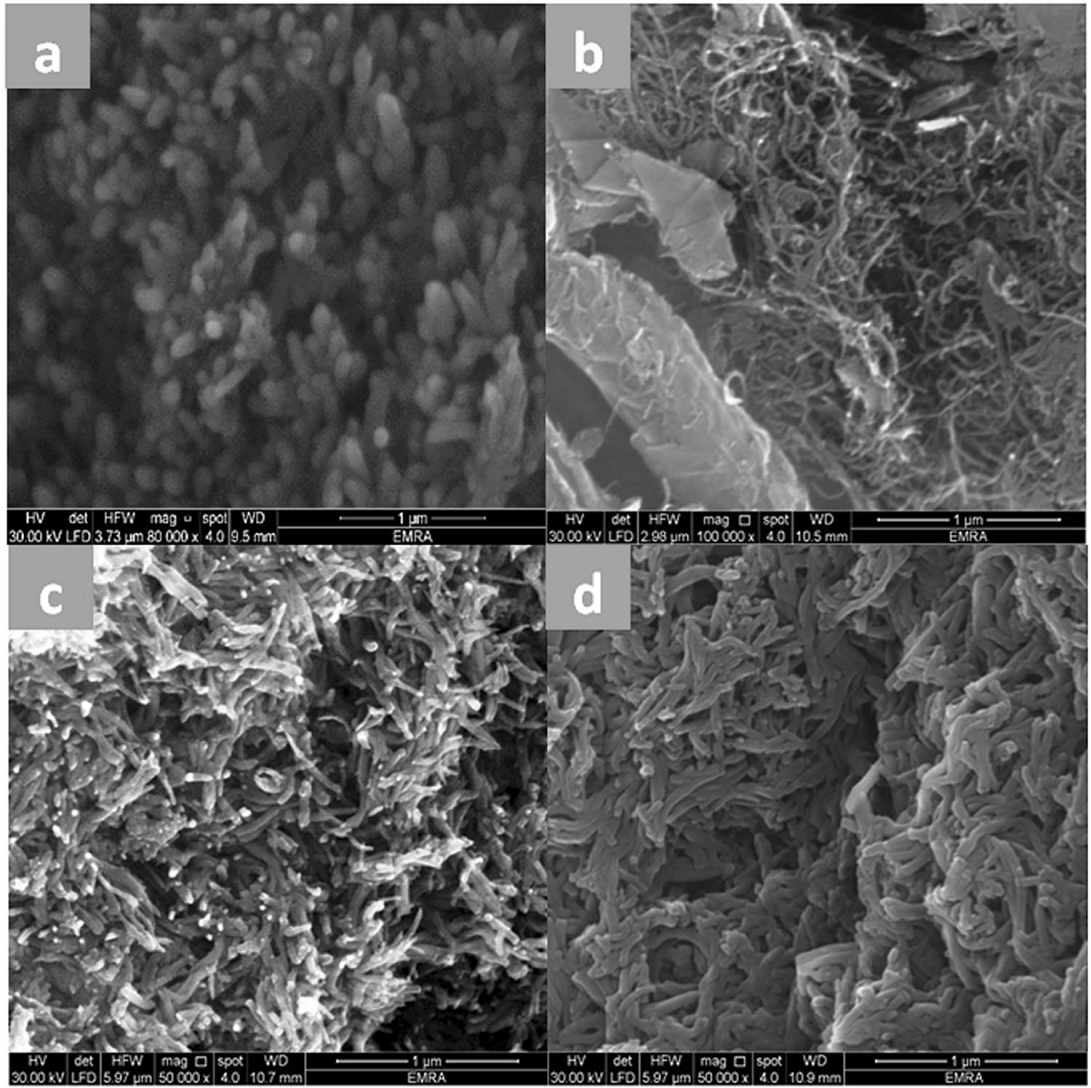
SEM images of CPA (**a**), N-SWCNTs-GO-CE (**b**), CPA/N-SWCNTs-GO-CE (**c**), and CPA/N-SWCNTs-GO-CE/CuO nanocomposites (**d**).

The surface morphologies of the CPA (a), N-SWCNT-GO-CE (b), CPA/N-SWCNT-GO-CE (c), and CPA/N-SWCNTS-GO-CE/CuO (d) nanocomposites were studied by field emission scanning electron microscopy, as displayed in Fig. 1.The SEM micrographs mainly focus on the morphological modifications,which can be noticed on the surface of the samples^43^. The SEM micrographs further provided evidence of the nanocomposite modification^44^. The morphological parts of the nanocomposites were studied using a Jeol JSM-5400 LV SEM experiment device. The surface of CPA in Fig. 1a displays coral reefs with small holes in the spongy form. N-SWCNT-GO-CE nanofiller SEM images (Fig. 1b) show rod-like particles. These rods are linear (Fig. 1b enlargement X = 100,000). The SEM images of the CPA/N-SWCNT-GO-CE composite displays a surface fabrication upon inundation of N-SWCNTs-GO-CE in its matrix, which is obvious in the enlargement (x = 50,000) in Fig. 1c. The SEM images illustrate the framework with the accumulation of N-SWCNTs-GO-CE on the CPA surface, which prevents many smallhales. In the SEM images of CPA/N-SWCNTS-GO-CE/CuO (Fig. 1d), CPAs coated on CuONPs, which are decorated with N-SWCNTS-GO-CE hybrid composites, form a unique core–shell network.

**Figure 2:**
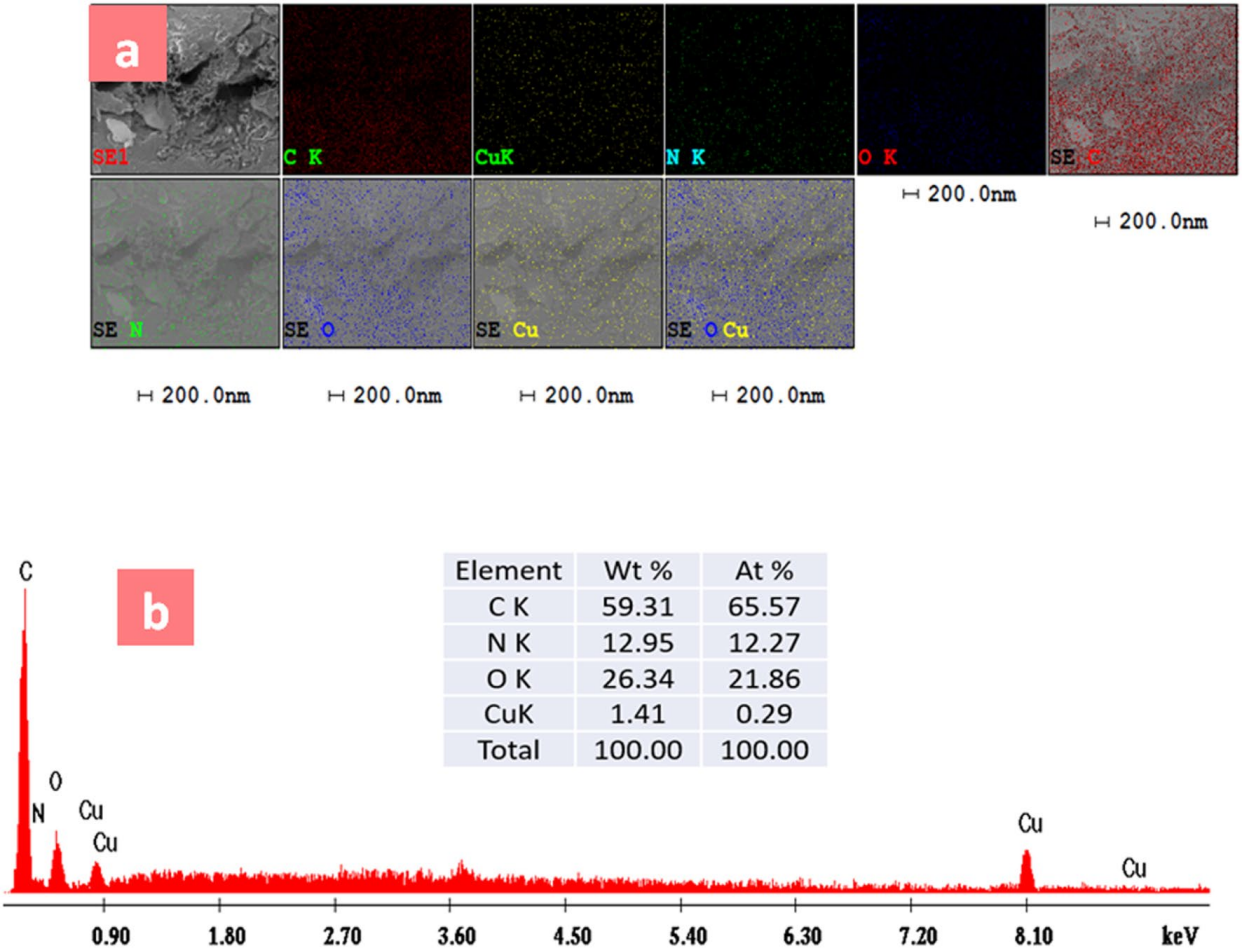
EDX (**a**) and maps analyzes (**b**) of CPA/N-SWCNTs-GO-CE/CuO nanocomposites.

Figure 2a displays the element mapping of C, N, O, and Cu, which is uniformly distributed in the nanocomposites and proves the presence of N-SWCNTs-GO-CE, CuO and CPA polymers. Figure 2b displays the EDX mapping for the CPA/N-SWCNT-GO-CE/CuO nanocomposites with the peaks of C, N, O, and Cu, which indicate the presence of CuO, N-SWCNTs-GO-CE, and CPA polymer.

**Figure 3:**
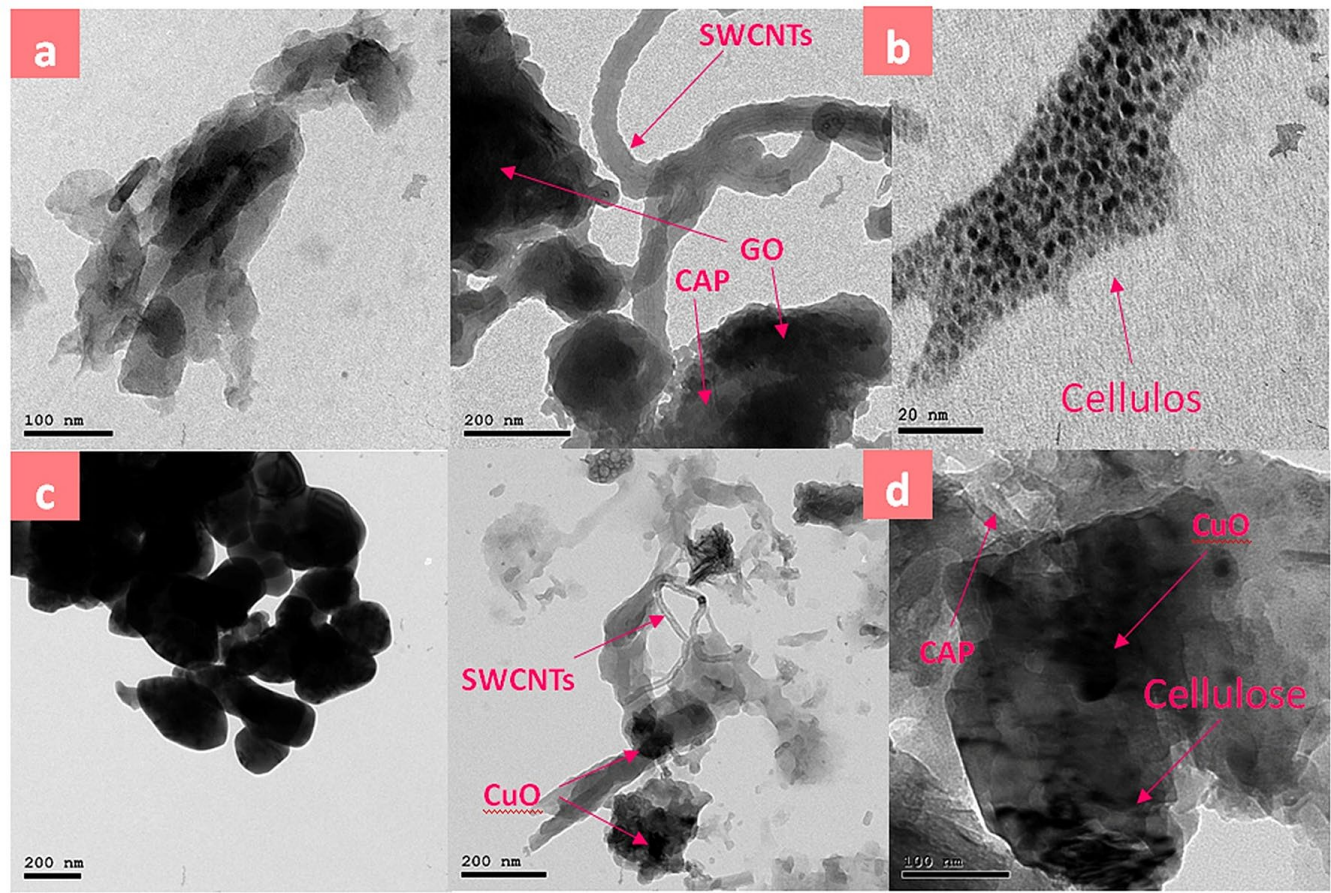
TEM images of CPA (**a**), CPA/N-SWCNTs-GO-CE (**b**), CuO (**c**), and CPA/N-SWCNTs-GO-CE/CuO nanocomposites (**d**).

Other morphological characteristics for CPA(a), CPA/N-SWCNT-GO-CE(b), CuO(c) and CPA/N-SWCNTS-GO-CE/CuO (d) nanocomposites are specified by TEM as illustrated in Fig. 3. The CPA image shows accumulated layers in Fig. 3a with a scale of 1 μm. TEM images of CPA/N-SWCNT-GO nanocomposites in Fig. 3b show that N-SWCNT-GO-CE nanofillers are instilled in the CPA polymer matrix and almost compose a pulp–shell structure. The black core is the GO nanoparticle, the rods are the SWCNTs; the smooth cortex indicate the polymer matrix. Cellulose nanofibres were decorated on the surface of the mixed NPs and matrix. In Fig. 3c, CuO shows a fibrous form. After N-SWCNTS-GO-CE was mixed with CuO, and CPA, the nanofillers are homogenously distributed and inserted in the CPA polymer, and the mixed N-SWCNTS-GO-CE/CuO are obviously immerged in the CPA polymer matrix (Fig. 3d).

### Thermal analysis

**Figure 4:**
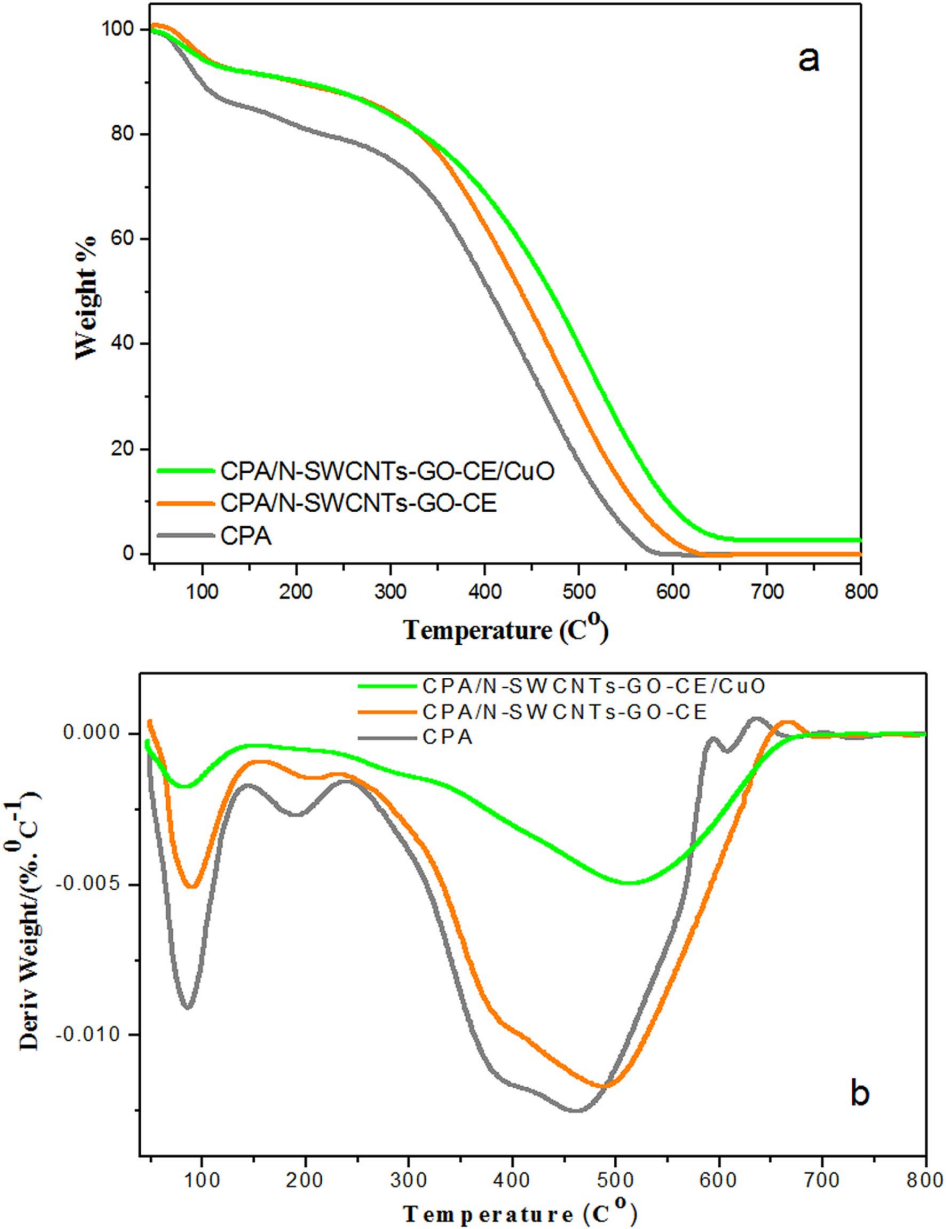
(**a**) TGA curves for pure CPA, CPA/N-SWCNTs-GO-CE and CPA/N-SWCNTs-GO-CE/CuO nanocomposites. (**b**) DTG curves for pure CPA, CPA/N-SWCNTs-GO-CE and CPA/N-SWCNTs-GO-CE/CuO nanocomposites.

The thermal attitudes of CPA, fabricated CPA/N-SWCNT-GO-CE, and CPA/N-SWCNTS-GO-CE/CuO nanocomposites were studied by TGA and DTG technique in air at a heating rate of 10 °C min^−1^, as displayed in Figs. 4a,b. The thermal analysis was performed in a temperature range of 90–800 °C. Table 1 displays the disintegration temperatures for various percentages. The temperatures for10, 25 and 50% weight losses are T_10_, T_25_ and T_50_, respectively. The TGA curve display slight weight losses in the range of 95–100 °C, which are more than 5% of the entire weight loss. This weight loss is attributed to the loss of solvents and absorption of moisture and/or water molecules^51,52^.

**Table 1:** Thermal behavior of CPA, CPA/N-SWCNT-GO-CE, and CPA/N-SWCNTS-GO-CE/CuO nanocomposites.

Products	CDT_*max*_ (°C)	CDT_*final*_ (°C)	Temperature (°C) for various percentage decompositions		
			T_10_	T_25_	T_50_
CPA	462.44	592.29	541.22	484.08	407.47
CPA/N-SWCNT-GO-CE	492.53	619.38	575.78	514.13	440.48
CPA/N-SWCNTS-GO-CE/CuO	522.35	658.46	614.87	548.69	475.04

Moreover, the TGA analysis of all examined samples shows the role of mixed N-SWCNTS- GO-CE/CuO nanocomposites on the thermal stability of CPA. The TG curves analysis also shows that the studied samples fundamentally decomposed in three decomposition steps. The decomposition begins early in all samples and remain at higher temperatures with decomposition weight loss percentages below 50%. This notice is completely referred to the presence of air atmosphere. The first decomposition step is fundamentally referred to the complete elimination of solvents^14^. The first step was finish at approximately 150 °C and is attributed to the chloride ions linked to the positive positions on the CPA chain, which referred chain degradation. The second decomposition step, which fundamentally occurred at a higher temperature, started at approximately 350 °C and completed at approximately 600 °C and is referred to additional decomposition of CPA chains to smaller chains^52^. Comparable observations are displayed for the CPA/N-SWCNT-GO-CE, and CPA/N-SWCNTS-GO-CE/CuO nanocomposites with small increases in the high temperatures, i.e., increased thermal stability.

Meanwhile, the addition of mixed N-SWCNTS-GO-CE/CuO increases the thermal stability of CPA, which indicates the presence of intermolecular interactions between nanofillers and polymer matrix. T_10_, T_25_ and T_50_ show important and gradual increases from CPA (the lowest) to CPA/N-SWCNTS-GO-CE/CuO (the highest). From the DTG analysis, the CDT_max_ amount is defined as the maximum temperature at which decomposition occurs^46^, i.e., the composite degradation temperature. These amounts are fully identical in all inspected nanocomposites and are approximately 492 °C ± 30.

### FT-IR analysis

**Figure 5:**
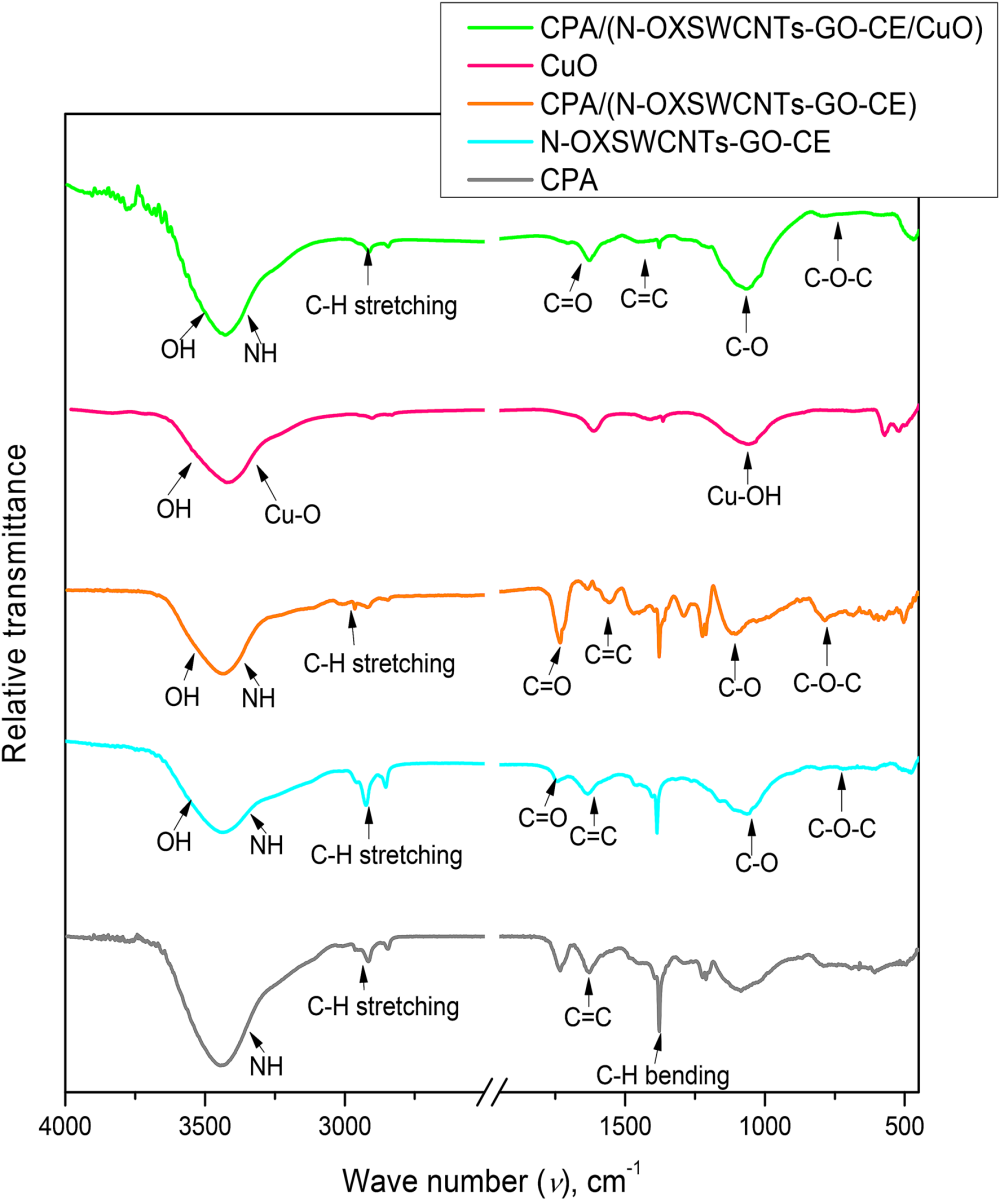
IR spectra for pure CPA, N-SWCNTs-GO-CE, CPA/N-SWCNTs-GO-CE, CuO, and CPA/N-SWCNTs-GO-CE/CuO nanocomposites.

An FT-IR spectroscopic analysis was investigated from 4000 to 400 cm^−1^. The acquired spectra for CPA, N-SWCNT-GO-CE, CuO, CPA/N-SWCNT-GO-CE and CPA/N-SWCNT-GO-CE/CuO in FT-IR are displayed in Fig. 5. The FT-IR spectrum of the CPA/N-SWCNT-GO-CE/CuO composite shows the bands associated with pure CPA and N-SWCNT-GO-CE/CuO peaks. In all spectra, broad bands at approximately 3,400 cm^-1^ are attributed to the N–H and C–H stretching modes^53,54^. The two N–H stretching modes are noticed as broad infrared bands with very large intensity (Fig. 5) and attributed to the presence of strong intermolecular H-bonding in molecules^55^. In pure CPA, CPA/N-SWCNT-GO-CE and CPA/N-SWCNT-GO-CE/CuO, a characteristic doublet of bands appears at 1627 and1736 cm^−1^ in the infrared spectrum of aniline and p-PDA^56^. The bands due to the C=O stretch are very prominently observed at 1745 cm^–1^ for the carboxylated SWNT. Other bands are a tiny one at 3452 cm^–1^ and another at 2952 cm^–1^, which indicate O–H and C–H stretches, respectively. The O–H vibration is related to amorphous carbon because amorphous carbon readily shapes a bond with atmospheric air, and there are C–C vibrations due to the internal disorder. An anti-symmetric stretch C–O is also obvious at 1660 cm^–1^. The bands at 1236 cm^–1^ and 1405 cm^–1^ indicate C=C^57^. In cellulose, the broad band in the 3602–3110 cm^-1^region is due to the OH-stretching vibration. The presence of amorphous cellulosic can be ascertained by the shift of the band from 2910 cm^-1^, which indicates the C–H stretching vibration. Furthermore, the FTIR absorption band at 1420 cm^-1^ is attributed to a symmetric CH_2_ bending vibration. The FTIR band at 896 cm^-1^ is attributed to C–O–C stretching^57^. The FT-IR spectra of nanocomposites also display distinguishing bands of GO. Broad bands at1732, 1624, and 3300–3615 cm^−1^ are referred to the C = O stretch of the carboxylic acid group, C=C, and O–H, respectively. The bands at 1226 cm^−1^ refer to the epoxy (C–O) ring stretching, and the bands at 642 cm^−1^refer to the symmetric ring disfigurement of the epoxy group on the GO nanofillers^15^. In the nanocomposites, there is no remarkable signal for CuO, since the assigned CuO is too small to be detected by FT-IR.

### XRD analysis

**Figure 6:**
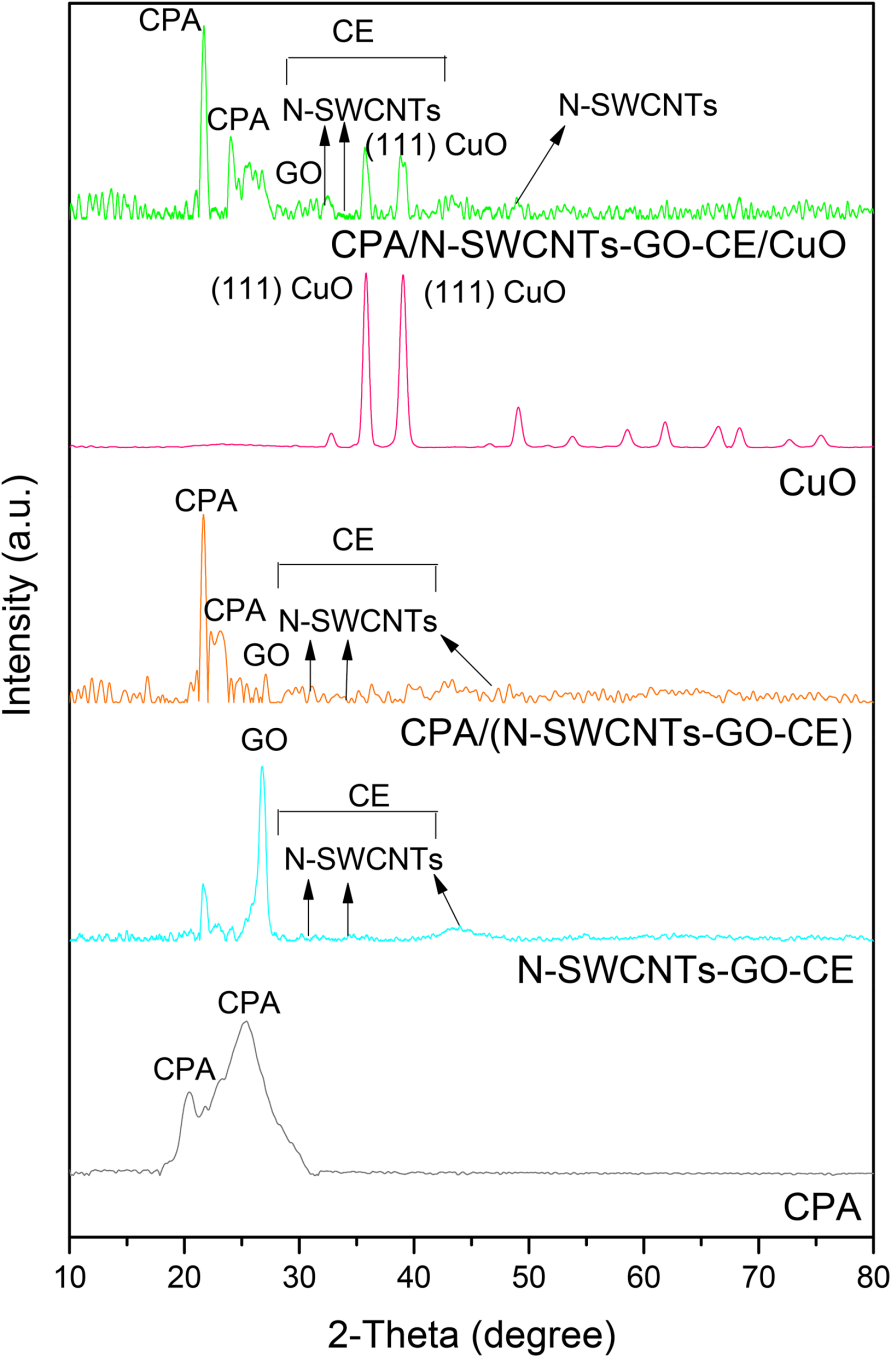
XRD diffraction patterns for pure CPA, N-SWCNTs-GO-CE, CPA/N-SWCNTs-GO-CE, CuO, and CPA/N-SWCNTs-GO-CE/CuO nanocomposites.

The expected shape of the prepared nanocomposites has been studied using X-ray diffraction techniques. The XRD characterization styles give a clear evidence for the composite fabrication. XRD diffractograms for CPA, N-SWCNT-GO-CE, CuO, CPA/N-SWCNT-GO-CE and CPA/N-SWCNT-GO-CE/CuO nanocomposites are shown in Fig. 6 over the measuring range of *2θ* = 10–80°.CPA and N-SWCNT-GO-CE/CuO nanocomposites physically react with each other via the nanocomposite production. The *2θ* scan displays the perfect X-ray diffraction patterns for CPA and mixed N-SWCNT-GO-CE/CuO. Figure 6 displays the XRD pattern for mixed N-SWCNT-GO-CE. Three intense peaks at 49°, 33°, and 31°can be ascribed to the crystalline region of N-SWCNTs^58^. Other distinguishing peaks near 30–40° refer to amorphous cellulose^59^. The diffraction peak with a maximum at approximately25° displays the distinguishing composition of GO^60^. For the CuO sample, sharp intense peaks at *2θ* of 35.8° and 39.1° conformable to the (111) plane were noticed with other less intense peaks characteristic of CuO.

However, the copolymer CPA displays the XRD patterns with two broad diffraction peaks at 2θ = 18.4° and 26.4°, which are typical of an amorphous substance^58,60^ and as illustrated in Fig. 6. These patterns demonstrate a weak degree of crystallinity, since no sharp crystalline beaks are noticed. All of the previous peaks related to the mixed N-SWCNT-GO-CE/CuO and CPA have been mentioned in the XRD pattern for CPA/N-SWCNT-GO-CE/CuO nanocomposites and illustrated in Fig. 6. The XRD diffraction patterns for CPA/N-SWCNT-GO-CE/CuO hybrid nanocomposites are notable with no confusion of the composition of competent nanocomposites. No other crystalline peaks may be specified to another stage or presence of muck in the desirable nanocomposites.

### Raman analysis

**Figure 7:**
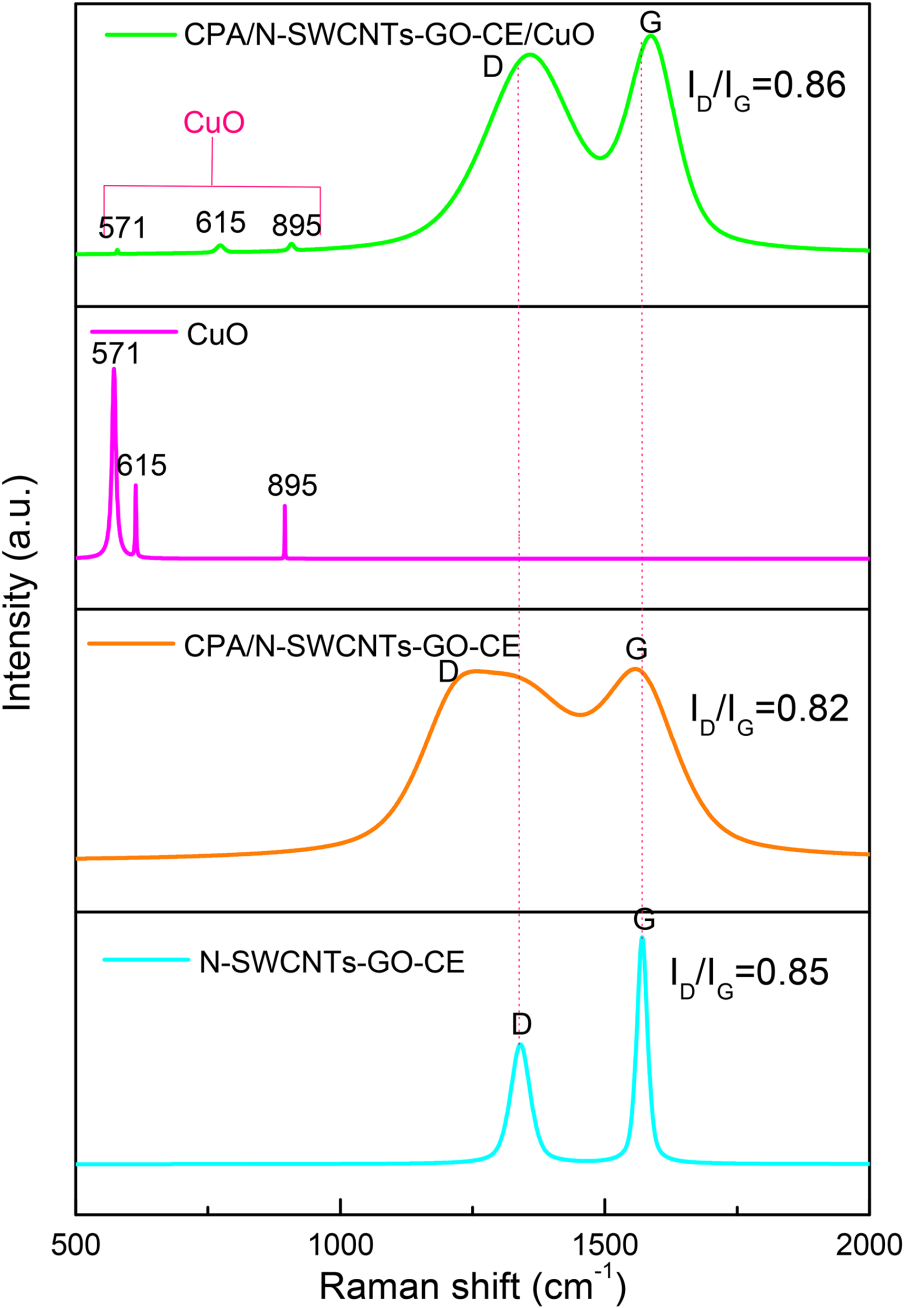
Raman spectrum of N-SWCNTs-GO-CE, CPA/N-SWCNTs-GO-CE, CuO and CPA/N-SWCNTs-GO-CE/CuOnanocomposites.

Raman spectroscopy is applied to prove the presence of N-SWCNTs-GO with CPA in nanocomposites. Figure 7 displays the Raman spectra of N-SWCNTs-GO-CE, CPA/N-SWCNTs-GO-CE, CuO and CPA/N-SWCNTs-GO-CE/CuO nanocomposites. The D band is disorder-induced, e.g., by sp3-hybridized carbon atoms, whereas the G band represents the in-plane stretching E_2g_ mode^61^. The distinctive properties in the Raman spectrum of N-SWCNTs-GO-CE are the so-called D band, which is located at approximately 1340 cm^−1^, and the G band is located at 1568 cm^−1^. The G and D peaks of N-SWCNTs-GO-CE appear in the Raman spectra of CPA/N-SWCNTs-GO-CE nanocomposites. The G and D bands of CPA/N-SWCNTs-GO-CE nanocomposites are slightlydifferent from those of N-SWCNTs-GO-CE and appear at 1571 and 1289 cm^−1^, respectively. The G and D bands of N-SWCNTs-GO-CE are also present at 1586 cm^−1^ and 1360 cm^−1^ in CPA/N-SWCNTs-GO-CE nanocomposites and shifted compared with those of N-SWCNTs-GO-CE. The behaviour of all nanocomposites demonstrates that the shift of the G band illustrates a strong interaction between N-SWCNTs-GO-CE and the polymer and possibly a charge transfer between N-SWCNTs-GO-CE and CPA. The expanded G band of nanocomposites mainly refers to the covering effect of amorphous CPA on N-SWCNTs-GO-CE during synthesis^62,63^.

The G and D band intensity ratio is a substantial factor to measure the graphitization degree in the required material. The variation in band intensity ratio (I_D_/I_G_) of the original N-SWCNTs-GO-CE and nanocomposites is a perfect indicator of the proportional grade of defects in N-SWCNTs-GO-CE. If both bands have comparable intensities, there is a high level of structural defects^64^. The I_D_/I_G_ ratios of CPA/N-SWCNTs-GO-CE, and CPA/N-SWCNTs-GO-CE/CuO nanocomposites are 0.85, 0.82 and 0.85. The CPA/N-SWCNTs-GO-CE/CuO nanocomposites has a higher I_D_/I_G_ ratio than N-SWCNTs-GO-CE, which suggests that no defects were created in the N-SWCNTs-GO-CE lattice. Moreover, the peaks at 577–907 cm^−1^ can be ascribed to CuO. These results confirm the presence of CuO in the synthesized CPA/N-SWCNTs-GO-CE/CuO nanocomposites. The Raman spectra of CuO show three peaks at 571 cm^−1^, 615 cm^−1^ and 895 cm^−1^.

### Mechanism of polymerization

**Figure 8:**
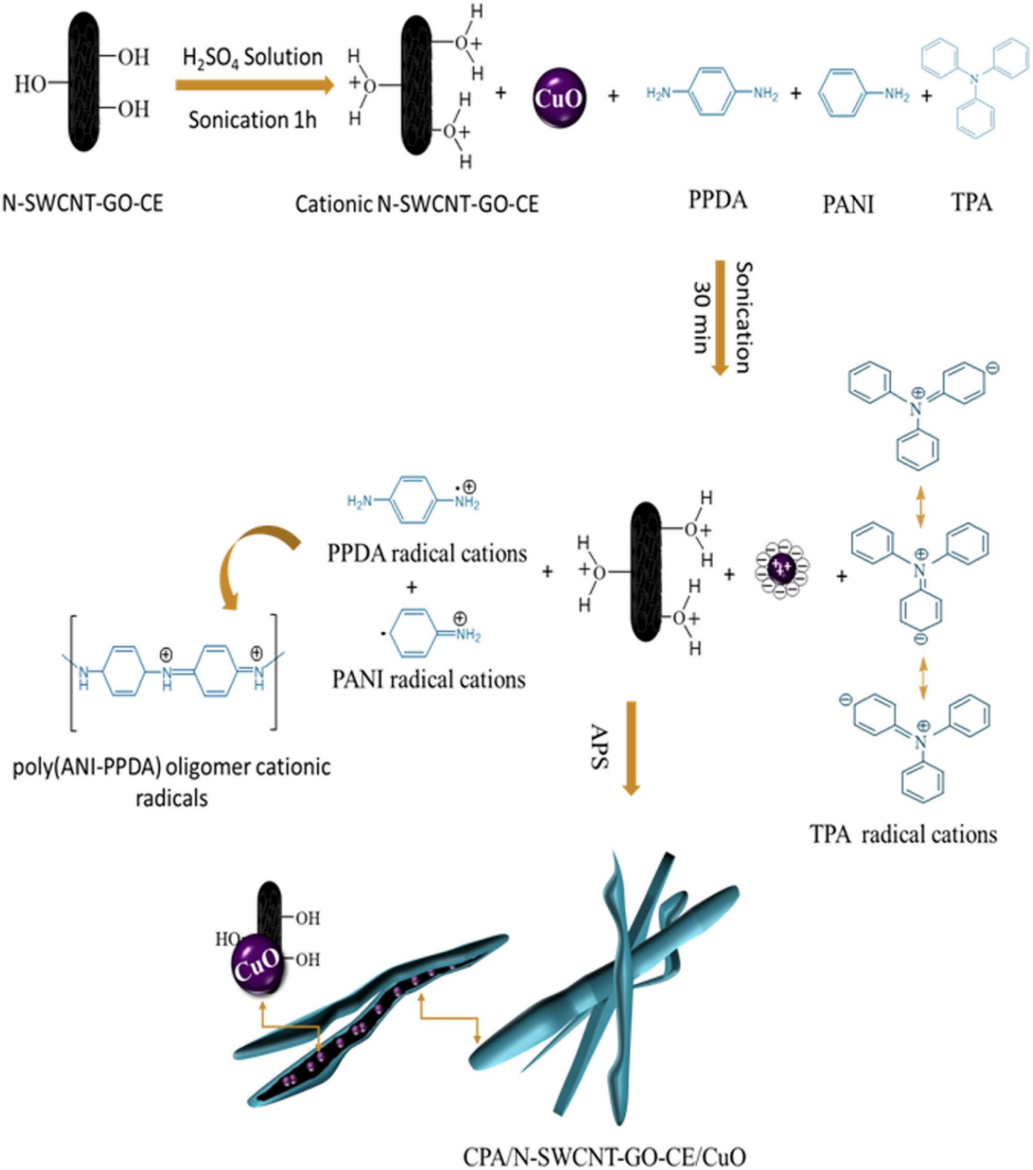
Illustration of the fabrication procedure of core–shell PAC/N-SWCNTs-GO-CE/CuO nanocomposites.

According to the investigated results, a suggested mechanismto construct such modern hybrid nanocomposite is proposed to illustrate the fabrication method of CPA/N-SWCNTs-GO-CE/CuO nanocomposite (Fig. 8). The CPA/N-SWCNTs-GO-CE/CuO nanocomposite was synthesized by the softcopolymerization procedures using the chemical oxidative technique. The polymerization occurs in the presence of ANI with TPA and PPDA as cross-linkers with N-SWCNTs-GO-CE in an acidic medium. In the acidic environment, the -COOH groups on the surface of N-SWCNTs-GO-CE becomebe protonated, i.e., they gain H^+^ from the medium^65^. Therefore, the adsorption of SO_4_^=^ anions may compensate for the positive charges on the N-SWCNTs-GO-CE surfaces and CuO NPs. Moreover, in the charge compensation method, additional adsorption of SO_4_^=^ on the N-SWCNTs-GO-CE and CuO NPs surface can work as the charge compensator for positively charged CPA chains in the formation of CPA/N-SWCNTs-GO-CE/CuO nanocomposite.ANI, PPDA and TPA are oxidized into cationic radicals under acidic environments using APS. Then, the polymerization of cationic radicals of ANI and PPDA creates poly(ANI-PPDA) as linear oligomers with two amino groupsis terminated.

The linear poly(ANI-PPDA) oligomer cationic radicals continue to react with TPA cationic radicals after oxidation tocompose a CPA network by polymerization at the three N para-positions due to similar reaction activities. Electrostatic interactions occur between cationic radicals withanions absorbed on both N-SWCNTs-GO-CE and CuO surface. Furthermore, it is conceivable that three types of hydrogen bonding occur: between chains of CPA and oxygen atoms on the N-SWCNTs-GO-CE surface,among the chains of CPA in the nanocomposite of CPA/N-SWCNTs-GO-CE/CuO, and between chains of CPA and oxygen atoms on the CuO surface. Finally, the π–π stacking between the π bonds of N-SWCNTs-GO-CE and the aromatic rings of CPA stabilizes the bound complex structure of the CPA/N-SWCNTs-GO-CE/CuO nanocomposite. The interactions can confirm that N-SWCNTs-GO-CE and CuO are inserted to produce core–shell structures and CPA chains^66–70^. Such above mentioned mechanism enhance the proposed charge transfer due to the use of such fabricated nanocomposites. The reported nanocomposited based on cross-linked PANI and coated with cellulose in the presence of mixed N-SWCNTs-GO nano-filler. In addition to the existence of CuO as well.

### UV–Vis spectroscopic measurement for MO dye removal and photocatalytic degradation

**Figure 9:**
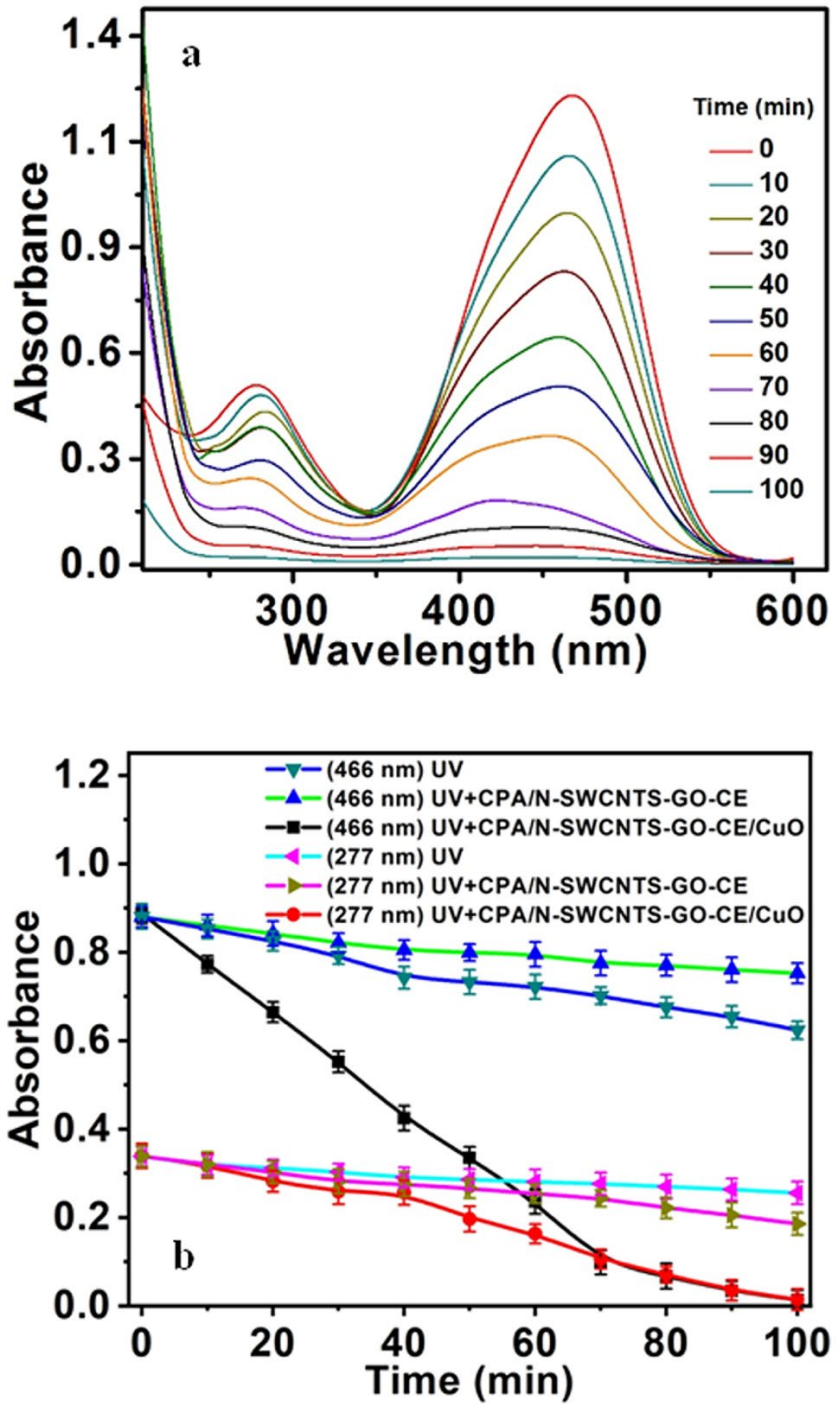
(**a**) photo-absorption spectral measurements of methyl orange in the presence of CPA/N-SWCNTs-GO-CE/CuO nano-catalysts using 50 mg/L nano-catalysts, MO 10 mg/L and at pH 6; (**b**) Alterations of the absorbance spectra versus time at wavelengths of 277 and 466 nm in three different cases UV radiation, in the presence of PCP-CNTs and nano-catalysts in presence of UV radiation.

**Figure 10:**
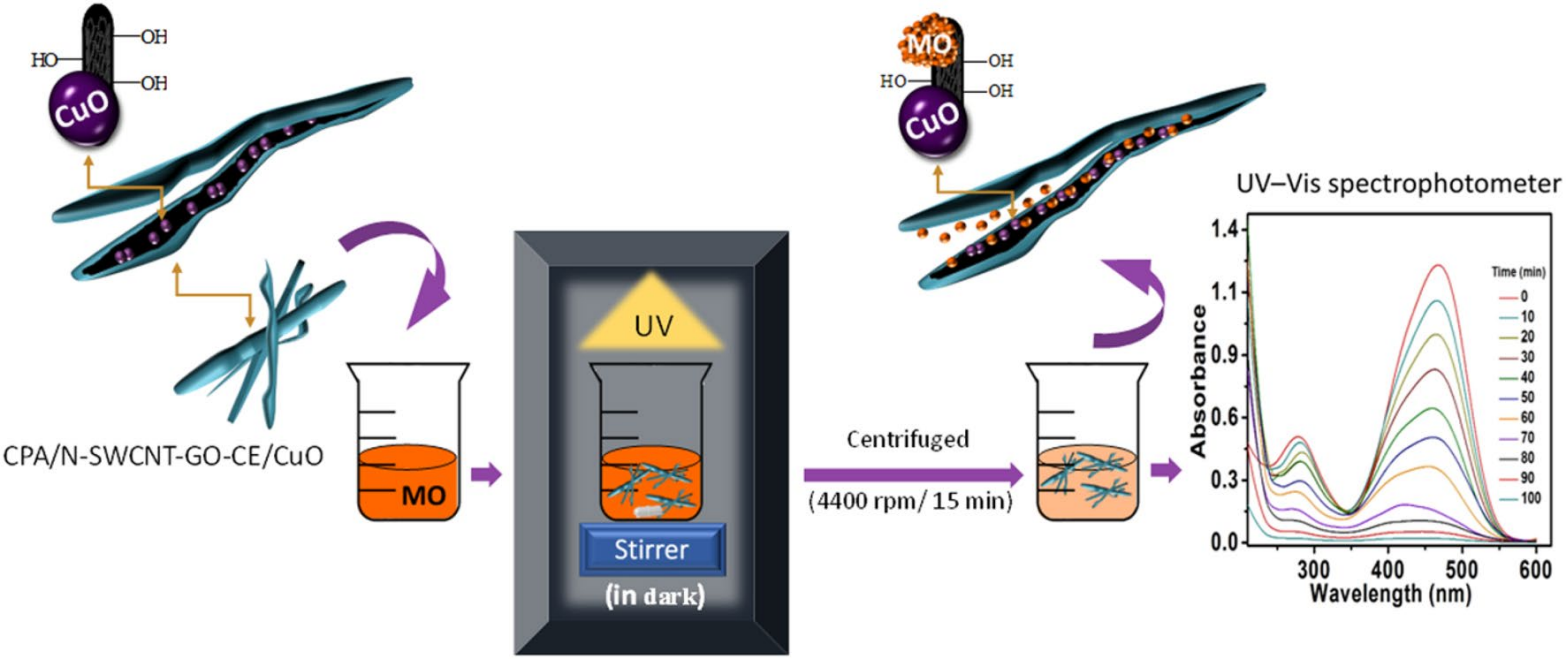
A smart overview for the photocatalytic degradation process og MO based on the CPA/N-SWCNTs-GO-CE/CuO nanocomposite.

MO has dual absorbance peaks at 277 and 466 nm. This dye shows high stability with distinguished absorption peaks in the absence of CPA/N-SWCNTs-GO-CE/CuO nanocatalyst. The rate of decolorization is approximately zero, and the photo-degradation doesnot entirely occur (see Fig. 9a). By using the CPA/N-SWCNTs-GO-CE/CuO nanocatalyst, we observe a substantial decrease in the main absorption peaks at 277 and 466. These changes are due tothe extensive mineralization of MO,which comprises the phenyl ring degradation. Furthermore, a considerable quenching at 466 nm occurs due to bleaching, which involves the azo bond cleavage (Fig. 9b). For additional investigation, the optical behaviour of the nanocatalyst-based CuO was examined using three conditions: under UV radiation, in the presence of (CPA/N-SWCNTs-GO-CE) and the combination of CPA/N-SWCNTs-GO-CE/CuO nanocatalyst under UV. The data analysis provides the photobleaching and degradation of the MO in the presence of the CPA/N-SWCNTs-GO-CE/CuO nanocatalyst under UV in the maximum time of 100 min. Moreover, the bleaching and photodegradation processes of MO completely disappear in the absence of the CPA/N-SWCNTs-GO-CE/CuO nanocatalyst. The results show that the adsorbability of MO on the nanocomposite surface does not change within 6 min after stirring the solution in the dark, and no further degradation occurs. Thus, with all conditions to investigate the adsorbability balance, the MO molecules and nanocomposite were stirred for approximately 10 min without light sources. Furthermore, Fig. 10 shows a smart summarized overview for the photocatalytic degradation process.

#### Effect of the solution pH on the catalytic process

**Figure 11:**
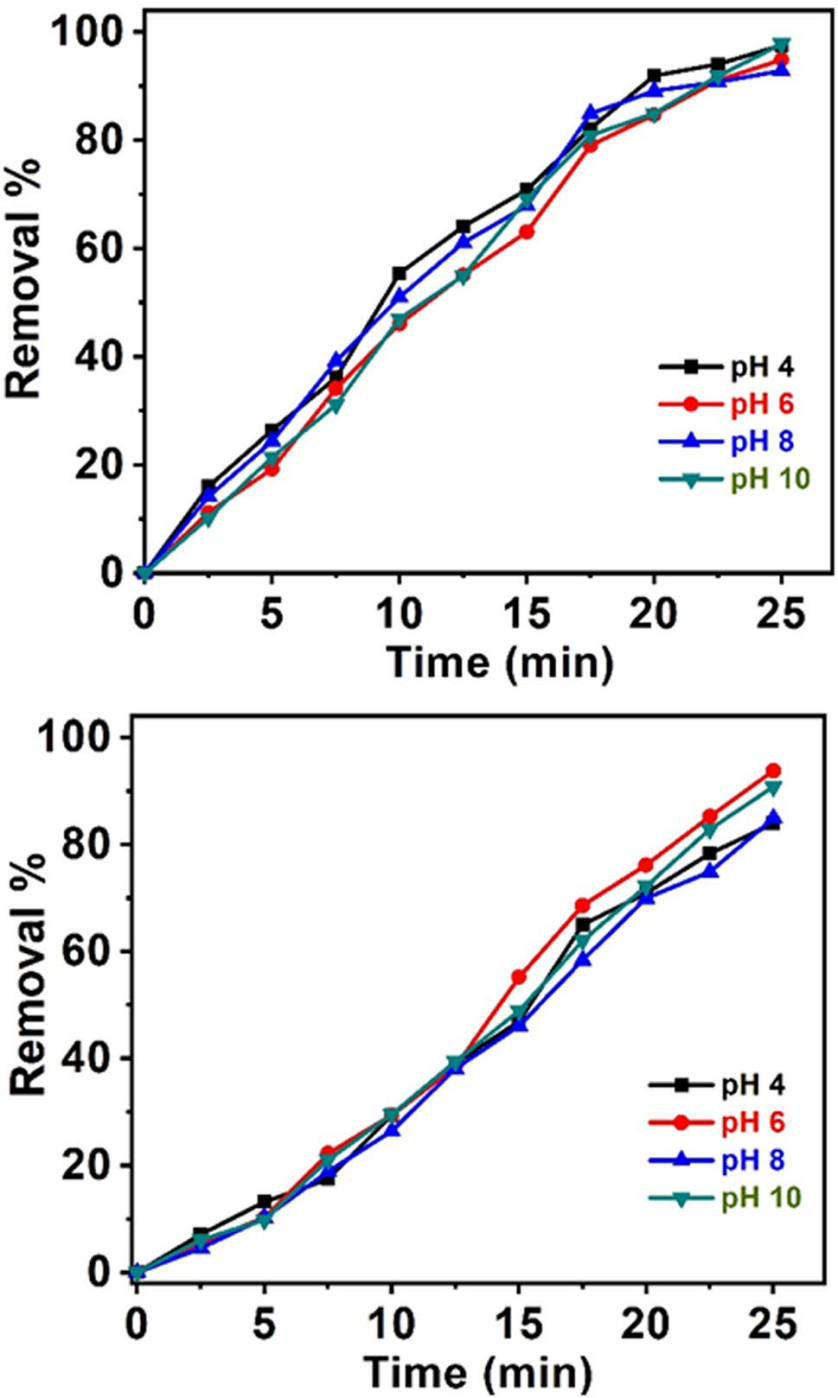
(**a**) pH effect on the bleaching process; (**b**) degradation efficiency of MO dye for 50 mg/L PCP-CNTs-Cu, MO 10 mg/L.

The photocatalytic experimentation of the pH effect was investigated at a range of pH 4–10 using 50 mg of nanocomposite in 100 mL MO dye solution of 10 mg/L concentration at adjusted contact time. Moreover, different pH solutions were obtained using standard solutions of hydrochloric acid and sodium hydroxide. Figure 11 shows the pH effect on the proficiency of MO degradation and bleaching. The highest degradation and bleaching rates are detected at pH 6 (Fig. 11). These pH significances show that the degradation percentage increases with increasing pH up to 6; then, itbegins to quench. Generally, the pH effect on the degradation depending on the nanocomposite has been assorted with the establishment of acid–base equilibria to monitor the chemical behaviour of the nanocomposite surface^71,72^. Therefore, all following experiments were performed at pH 6. The pH effect on the adsorption and removal proficiency can be ascribed to the dissimilarity of Coulomb interactions between the CuO-based nanocomposite surfaces and MO. These interactions tend to enhance the adsorbability of MO molecules to the nanocomposite surfaces^73^ and quench in the acidic medium due to the driving forces^74^.

Additionally, MO has a pKa value of approximately 3.8^75^. In excess of this pKa and with increased pH, MO provides anions, which make it more easily bind to the surface of the CuO-nanocomposite. Thus, when the pH of the medium increases to 6, the adsorbability and removal efficiencies increase. At pH > 6, the bleaching and degradation were enhanced, which can be attributed to the quenching of the oxidation potential of hydroxyl radical due to increased pH^76^. However, the increase in hydroxyl ions at pH > 6 fights with MO anions at the nanocomposite surface^77^.

#### Effect of the CPA/N-SWCNTs-GO-CE/CuO nanocomposite dose

**Figure 12:**
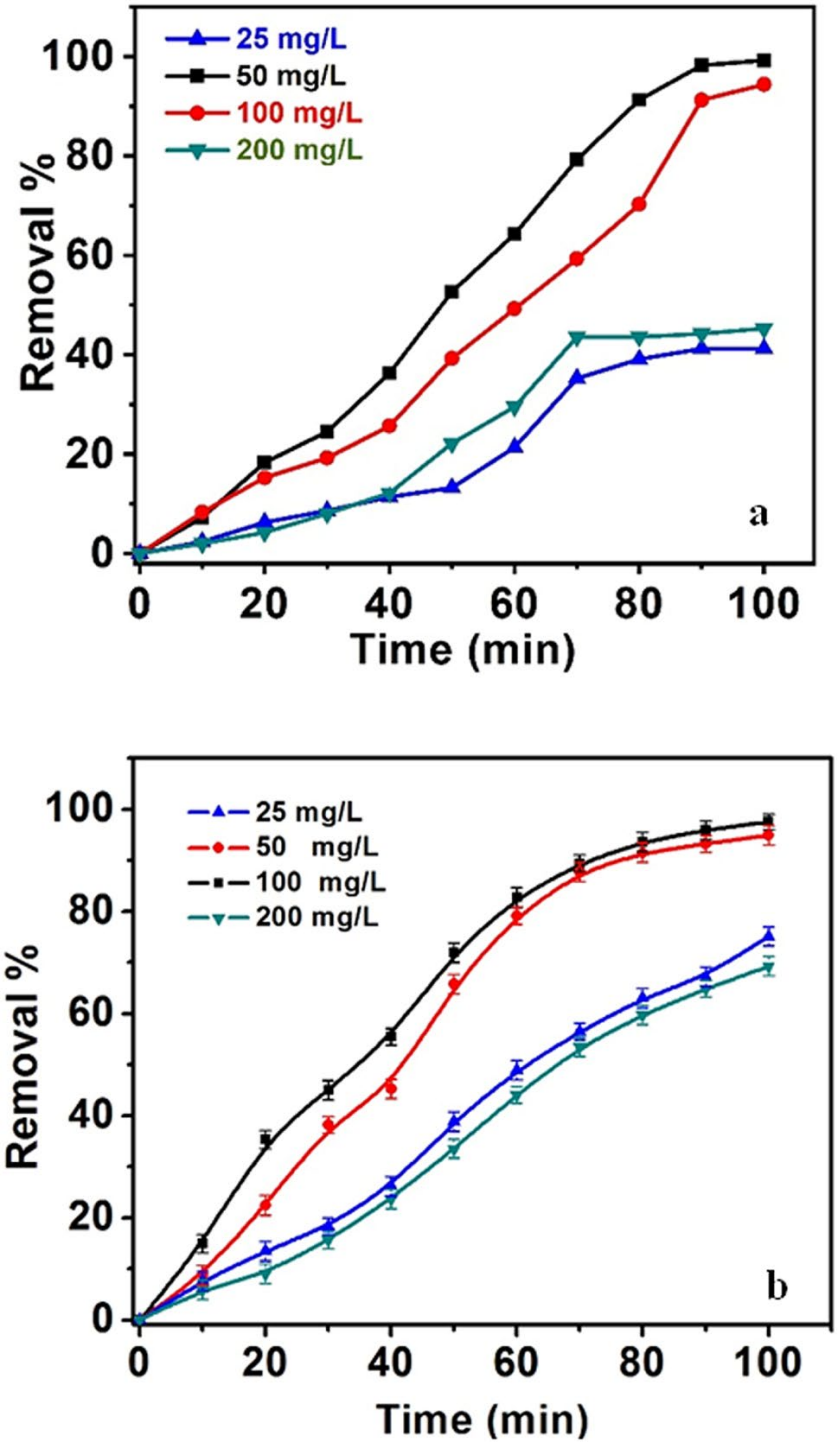
(**a**) Effect of CPA/N-SWCNTs-GO-CE/CuO nanospheres amount on the bleaching; (**b**) MO degradation, concentration of MO of 10 mg/L at pH 6.2 and time 100 min.

To investigate the effect of the nanocomposite dosage on the efficiency of bleaching and degradation of MO, different dosages of nanocomposite (25, 50,100 and 200 mg/L) were mixed with a solution containing 10 mg/L MO at pH 6 for 100 min. The obtained results are shown in Fig. 12. The data emphasize that the bleaching and degradation efficiencies increased with increasing nanocomposite content to 50 mg/L, exhibited no notable alteration when the nanocomposite weight increased to 100 mg/L, and extinguished when the nanocomposite quantity was 200 mg/L. This behaviour can be assigned to the presence of active sites, which increased with the increase in nanocomposite quantity. Consequently, the produced hydroxyl radicals enhanced the photocatalytic proficiency of the CuO-nanocomposite. With excess nanocomposite, the presence of a viscous colloid medium prevents UV light from penetrating the nanocomposite surface. Therefore, this effect inhibits the production of hydroxyl radicals and decreases the adeptness of MO deterioration and staining of the medium^78,79^. Thus, the nanocomposite quantity was adjusted to be 50 mg/L for further experimentation.

#### *Effect of C*_*i*_* of MO*

**Figure 13:**
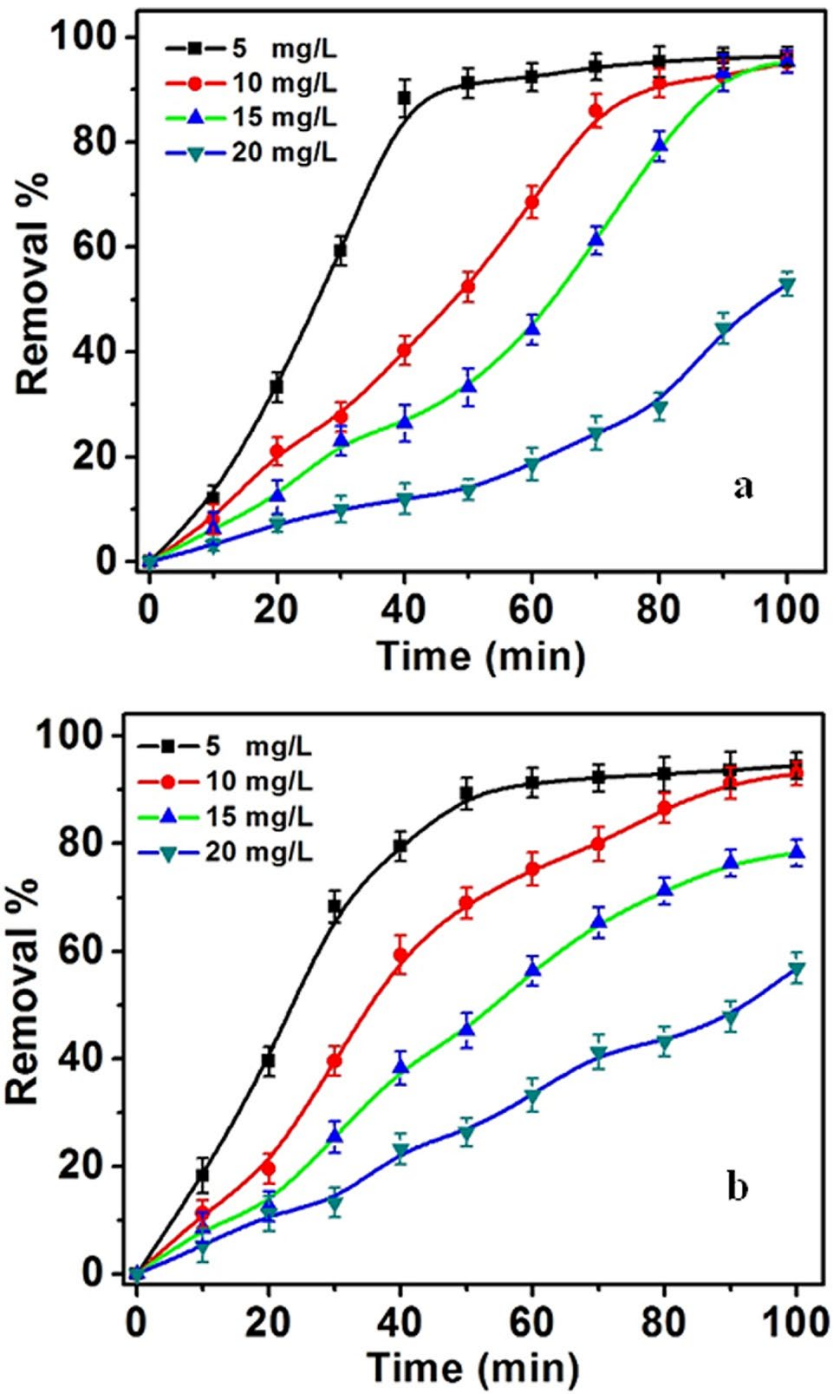
(**a**) Effect of initial concentration of MO on the bleaching; (**b**) degradation, the concentration of CPA/N-SWCNTs-GO-CE/CuO of 50 mg/L, pH 6.2 and time 100 min.

To investigate the effect of MO dye on the degradation process, MO concentrations were utilized in the range of 5–20 mg/L throughout a reaction time of 100 min at pH of 6. In addition, the amount of nanocomposite is 50 mg/L. The results are shown in Fig. 13. The obtained data demonstrate that the bleaching and degradation decreased with increasing *C*_*i*_ of MO. This result can be related to the decrease in number of active sites of the CPA/N-SWCNTs-GO-CE/CuO nanocatalyst surface. Thus, the introduction of hydroxyl radicals quenches and reduces the competence of the photo-catalytic reaction. Meanwhile, the increase in *C*_*i*_ of MO reduces the photon path length that diffuses through the MO medium. With more MO molecules, the dye can absorb significantlymore light than the CPA/N-SWCNTs-GO-CE/CuO nanocomposite, which reduces the competence of the photo-catalytic degradation process^80,81^. Thus, the suitable *C*_*i*_ of MO is 10 mg/L.

#### Oxygen content

**Figure 14:**
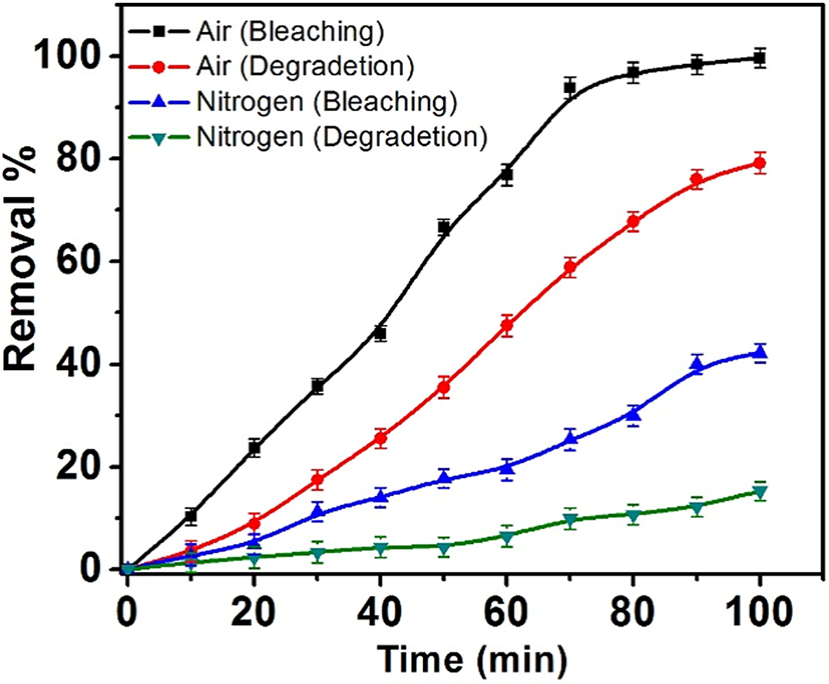
Oxygen effect on the rates of bleaching and degradation of MO at (time 100 min, pH = 6.2, the MO concentration of 10 mg/mL).

The effect of oxygen was investigated on both bleaching and degradation of MO based on the CPA/N-SWCNTs-GO-CE/CuO nanocomposite surface. The studied solution was conducted to air and nitrogen atmosphere to study the oxygen effect on the degradation process. The experimentations were accomplished by monitoring several parameters: time of 100 min, pH 6, *C*_*i*_ of MO 10 mg/mL, CPA/N-SWCNTs-GO-CE/CuO nanocomposite concentration of 50 mg/mL, and the solution was exposed to nitrogen gas for 2.5 min. Figure 14 shows the oxygen gas effect on the rates of bleaching and degradation. Furthermore, the analysis of the obtained results indicates that the adequacy of bleaching and degradation enhances with oxygen. This resultcan be attributed to the introduction of reactive species including hydroxyl radicals, oxygen radical and hydrogen peroxide^82^. Thus, the resulting radicals increase the efficiency of bleaching and degradation of MO.

#### The proposed photocatalytic degradation mechanism

**Figure 15:**
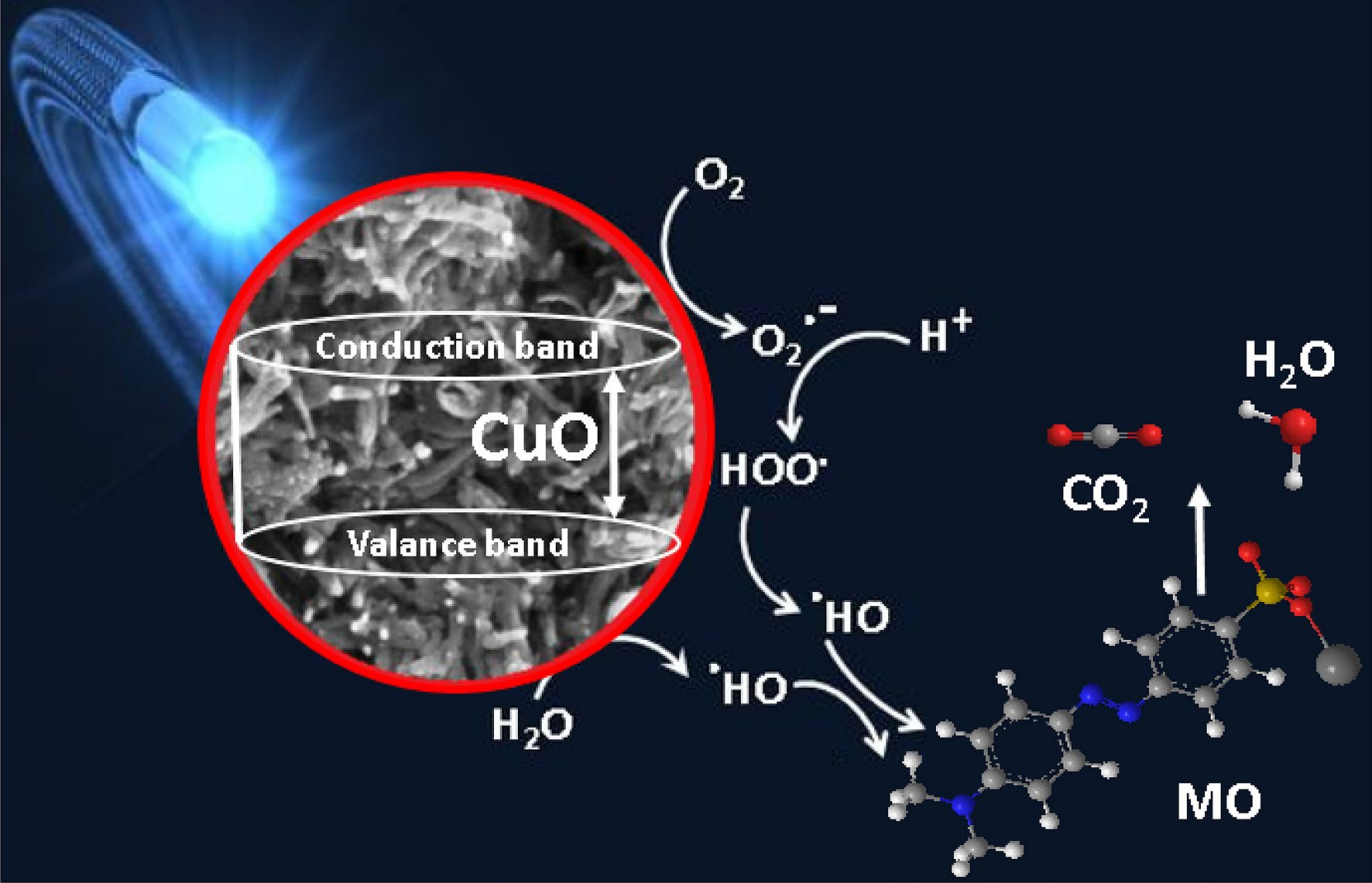
Reversibility of CPA/N-SWCNTs-GO-CE/CuO particles based on the bleaching and degradation rates of MO dye.

Schematic illustration explains the expected photocatalytic degradation mechanism for CPA/N-SWCNTs-GO-CE/CuO nanocomposite against MO dye has been given shortly in Fig. 15. Such photocatalytic decomposition of the azo dye on the CuO nanoparticles doped CPA/N-SWCNTS-GO-CE nanocatalysts under the UV radiation is presented in the following Fig. 15. Typically, the UV-radiation induces the valance band electrons of the CuO nanocatalysts convey to the conduction band. This equivalent energy is higher than the band gap of the doped CuO nanoparticles (2.43 eV)^83^, thus disseminating the initiation electrons (e^−^) of the conduction band and holes (h^+^) of the valance band. The oxidation of the azo dyes could be presented straightforwardly by the generated holes or by the distinctive interaction of these holes with (OH^−^) or H_2_O to create hydroxyl radicals (OH·). Also, the induced electrons reduce oxygen molecules (O_2_) adsorbed on the CPA/N-SWCNTS-GO-CE/CuO nanocomposite surface into superoxide (${\text{O}}_{2}^{ - }$) radicals. Finally, azo dyes were degraded by the induced OH· and ·${\text{O}}_{2}^{ - }$^84^. The significant interaction formulations are exhibited as follows:

$$ \begin{aligned} & {\text{nanocomposite doped CuO }} + hv \to {\text{h}}^{ + } + {\text{ e}}^{ - } \\ & \left( {{\text{OH}}^{ - } } \right){\text{ or H}}_{{2}} {\text{O }} + {\text{ h}}^{ + } \to {\text{OH}} \cdot \\ & \left( {{\text{O}}_{{2}} } \right) \, + {\text{ e}}^{ - } \to \cdot {\text{O}}_{2}^{ - } \\ & {\text{OH}}\cdot{\text{ and }}\cdot{\text{O}}_{2}^{ - } + {\text{ Azo dyes}} \to {\text{CO}}_{{2}} + {\text{ H}}_{{2}} {\text{O}} \\ \end{aligned} $$

In fact, the photo degradation process of the azo dye is mainly based on the chemical decomposition of MO molecules in the presence of ·${\text{O}}_{2}^{ - }$^83,84^.

#### Reversibility of the CPA/N-SWCNTs-GO-CE/CuO nanocatalyst

**Figure 16:**
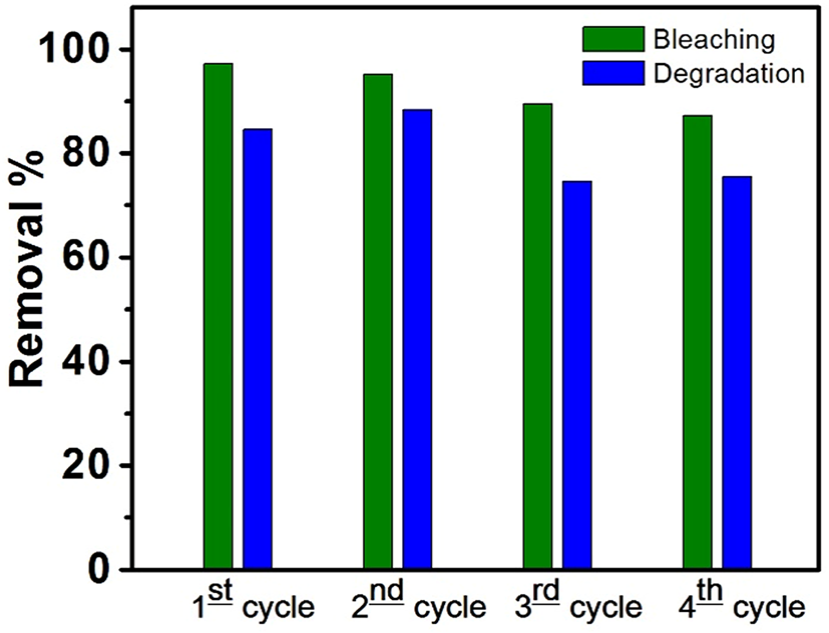
The proposed photodegradation mechanism of MO based CPA/N-SWCNTS-GO-CE/CuO under UV-light irradiation.

The reversibility of the CPA/N-SWCNTs-GO-CE/CuO nanocatalyst has a noteworthy effect from the economic viewpoint. Therefore, it is essential to study the competence of the nanocatalyst throughout the recovery process. Thus, the used CPA/N-SWCNTs-GO-CE/CuO nanocomposite was collected after the bleaching and degradation processes using a centrifuge with an adapted rate of 4400 rpm. Then, the centrifuged nanocatalyst yield was washed three times with ethanol and bi-distilled water. The separated nanocatalyst was dried and reused in more processes as shown in Fig. 16. The effectiveness of the MO bleaching and degradation reduce with the amount of recovered CPA/N-SWCNTs-GO-CE/CuO catalysts. In addition, the quenching of the degradation rate is elevated than the bleaching process.

Furthermore, comparable studies of using different adsorbent against methyl orange removal at the optimum conditions have been given in Table 2. The results confirm the extreme higher efficiency of CPA/N-SWCNTs-GO-CE/CuO nanocomposite compared to the other reported materials.

**Table 2:** Comparable studies of using different adsorbent in methyl orange removal at the optimum conditions.

Adsorbent	Temperature	Time	pH	Efficiency	Ref
Biochar from pomelo peels	25 °C	70 min	3	–	^85^
Activated carbon from shaddock peels	25 °C	150 min	3	54.25%	^86^
poly(3,4-ethylene dioxythiophene) (PEDOT)-modified polyvinylidene fluoride electrospun fibers	50 °C	360 min	3	75%	^87^
ZIF-67@CoAl-LDH	25 °C	90 min	3	72.3%	^88^
MnFe_2_O_4_@CPB/H_2_O_2_/visible LED light	25 °C	150 min	3	99.5%	^89^
Gd_2_O_3_/Bi_2_O_3_@GO	35 °C	45 min	6	95%	^90^
CPA/N-SWCNTS-GO-CE/CuO + UV light	25 °C	100 min	6	100%	Current study

## Conclusion

In this work, a novel CPA/N-SWCNTS-GO-CE/CuO nanocomposite was successfully prepared via an oxidative chemical polymerization method. The core–shell structure was clearly visualized through TEM, SEM, XRD, FT-IR and RAMAN studies. These synthesized nanocomposites were utilized to remove a hazardous dye (MO) and found to be highly efficient in its removal. Various parameters that affect the adsorption process, including *C*_*i*_ of MO, pH and dosage of CuO-based nanocomposite, were optimized. In addition, the oxygen content has a significant effect on the degradation process. The obtained data clarified that the dye was successfully degraded in the presence of CPA/N-SWCNTS-GO-CE/CuO compared to CPA/N-SWCNTs-GO-CE under UV radiation, which indicates the enhanced effect of CuO as a reinforced agent. The degradation process was investigated under the optimal conditions of pH of 6, 50 mg/L of CPA/N-SWCNTS-GO-CE/CuO photocatalyst, *C*_*i*_ of MO of 10 mg/L and under stirring at room temperature in atmosphere. This high efficiency of the CuO NP-based nanocomposite as a photocatalyst may provide a promising application for the degradation of dyes from aqueous solutions. In addition, the CPA/N-SWCNTS-GO-CE/CuO catalyst exhibits significant reversibility and highly recovered by effortless methods. This CuO-based photocatalyst provide an effective procedure with considerable effect in the wastewater treatment from dye pollution. Therefore, the present study has shown a simple and facile way for the synthesis of Cu NPs for the treatment of wastewater problems.

## Novelty of the work

The authors confirm this research work is novel and has not been published or sent for publication in another journal.
