# Author Correction: Network structure-based decorated CPA@CuO hybrid nanocomposite for methyl orange environmental remediation

**DOI:** 10.1038/s41598-021-99791-y

**Published:** 2021-10-07

**Authors:** Dina F. Katowah, Sayed M. Saleh, Sara A. Alqarni, Reham Ali, Gharam I. Mohammed, Mahmoud A. Hussein

**Affiliations:** 1 Department of Chemistry, Faculty of Applied Science, Umm Al-Qura University, P.O. Box 16722, Makkah, 21955 Saudi Arabia; 2 Department of Chemistry, College of Science, Qassim University, Buraidah, 51452 Saudi Arabia; 3 Chemistry Branch, Department of Science and Mathematics, Faculty of Petroleum and Mining Engineering, Suez University, Suez, 43721 Egypt; 4 Department of Chemistry, College of Science, University of Jeddah, Jeddah, Saudi Arabia; 5 Department of Chemistry, Faculty of Science, Suez University, Suez, 43518 Egypt; 6 Department of Chemistry, Faculty of Science, King Abdulaziz University, Jeddah, 21589 Saudi Arabia; 7 Polymer Chemistry Lab, Chemistry Department, Faculty of Science, Assiut University, Assiut, 71516 Egypt

Correction to: *Scientific Reports* 10.1038/s41598-021-84540-y, published online 03 March 2021

The original version of this Article contained errors.

Affiliation 2 was incorrectly given as ‘Department of Chemistry, Science College, Qassim University, Buraidah, Saudi Arabia’. Additionally, an affiliation was omitted for both Sayed M. Saleh and Reham Ali. The correct affiliations are listed below:

Affiliation 1:

Department of Chemistry, Faculty of Applied Science, Umm Al-Qura University, P.O. Box 16722, Makkah, 21955, Saudi Arabia

Dina F. Katowah & Gharam I. Mohammed

Affiliation 2:

Department of Chemistry, College of Science, Qassim University, Buraidah, 51452, Saudi Arabia.

Sayed M. Saleh & Reham Ali

Affiliation 3:

Chemistry Branch, Department of Science and Mathematics, Faculty of Petroleum and Mining Engineering, Suez University, 43721, Suez, Egypt.

Sayed M. Saleh

Affiliation 4:

Department of Chemistry, College of Science, University of Jeddah, Jeddah, Saudi Arabia

Sara A. Alqarni

Affiliation 5:

Department of Chemistry, Faculty of Science, Suez University, 43518 Suez, Egypt

Reham Ali

Affiliation 6:

Department of Chemistry, Faculty of Science, King Abdulaziz University, Jeddah, 21589, Saudi Arabia

Mahmoud A. Hussein

Affiliation 7:

Polymer Chemistry Lab, Chemistry Department, Faculty of Science, Assiut University, Assiut, 71516, Egypt

Mahmoud A. Hussein

The original Article has been corrected.

